# Perinatal exposure to gaseous pollutants and autism spectrum disorder in children: A nested case–control study in the nurses’ health study ii cohort

**DOI:** 10.1007/s11356-026-37583-5

**Published:** 2026-03-10

**Authors:** Hayon Michelle Choi, Bethsaida Cardona, Marianthi-Anna Kioumourtzoglou, Francine Laden, Jaime Hart, Marc G. Weisskopf

**Affiliations:** 1 Harvard University T H Chan School of Public Health, Cambridge, MA USA; 2 Graduate School of Green Growth & Sustainability, Korea Advanced Institute of Science and Technology, Daejeon, Daejeon, Republic of Korea; 3 Department of Epidemiology, Harvard TH Chan School of Public Health, Boston, MA USA; 4 Department of Environmental Health Sciences, Columbia University Mailman School of Public Health, New York, NY USA; 5 Channing Division of Network Medicine, Department of Medicine, Brigham and Women’s Hospital, Harvard Medical School, Boston, MA USA; 6 Boston, MA 02115 USA

**Keywords:** Maternal exposure, Carbon monoxide, Ozone, Nitrogen dioxide, Sulfur dioxide, Neurodevelopmental disorder

## Abstract

Our objective was to explore the association between maternal exposure to gaseous air pollutants and the odds of ASD in their child. We conducted a nested case–control study of participants in the Nurses’ Health Study II (NHS II) children born 1990–2002 with ASD (n = 250) and without ASD (n = 1,539) were randomly selected using frequency matching for birth years. The monthly average for each gaseous pollutant O_3_, NO_2_, CO, and SO_2_ were obtained from the daily values from the nearest 5 monitoring stations using distance-weighted averages from the nurse’s residence and averaged across months for different exposure windows. Logistic regression models were used to estimate adjusted odds ratios (OR) and 95% confidence intervals (CI) of ASD. Per interquartile range (IQR) increase for each pollutant from mutually adjusted models were: O_3_ in the 2nd trimester (OR per 0.007 ppm: 1.20; 95% CI: 0.96–1.50); SO_2_ in the 9 months before pregnancy (OR per 5.54 ppb: 2.26; 95% CI: 1.33–3.83); NO_2_ in the 3rd trimester (OR per 9.9 ppb = 2.10; 95% CI: 1.03–4.28); and CO in the 3 months after pregnancy (OR per 0.52 ppm: 1.36; 95% CI: 0.71–2.63). Our findings suggest different critical windows of exposure around pregnancy for different air pollutants.

**Supplementary Information:**

The online version contains supplementary material available at 10.1007/s11356-026-37583-5.

## Introduction

Autism spectrum disorder (ASD) is a complex neurodevelopmental condition marked by a deficiency in social communication and interaction, accompanied by repetitive behavioral patterns (American Psychiatric Association and Association 2013). The prevalence of ASD is steadily increasing in the U.S., from 6.7 per 1000 children aged 8 years (1 in 150 children) in 2000 to 27.6 per 1000 children (1 in 36 children) in 2020 (Prevention). ASD is influenced by various social and environmental factors across the lifespan.(Lyall et al. 2014; Robinson et al. 2016) There is a genetic contribution to ASD, however studies suggest the importance of environmental factors and possible interactions with multiple genes (Glasson et al. 2004; Hallmayer et al. 2011).

Air pollution is a mixture of various toxicants that can induce maternal systemic oxidative stress and pro-inflammatory cytokine production,(Hertz-Picciotto et al. 2005; MohanKumar et al. 2008) and have been associated with neurotoxicity to the fetus (Crump et al. 1998; Grandjean and Landrigan 2006). Such exposures could disrupt the fetal brain and alter the neonatal immune system, and in turn, be related to ASD. Different types of air pollutants are contributed from various sources, such as fine particulate matter (aerodynamic diameter ≤ 2.5 µm [PM_2.5_]) and sulfur dioxide (SO_2_) from combustion of fuel or wood, nitrogen dioxide (NO_2_) from traffic sources, ozone (O_3_) from chemical reactions between pollutants from vehicles and factories, and carbon monoxide (CO) from incomplete burning of carbon (Lin et al. 2019). Motor vehicles generate enormous quantities of CO, NO_2_, and PM_2.5_ which cause environmental pollution (Lin et al. 2019). The majority of prior work on air pollutants and ASD has focused on PM_2.5_ (Chun et al. 2020) and NO_2_ (Pagalan et al. 2019), with somewhat fewer examining O_3_ (Becerra et al. 2013; Dutheil et al. 2021). Far fewer studies have explored other air pollutants, including the other criteria pollutants SO_2_ and CO. Further, few studies have accounted for multiple exposure periods (pre-pregnancy, during pregnancy, and post-pregnancy) simultaneously to limit confounding by other exposure periods to better identify critical windows of exposure (Weisskopf et al. 2015).

The goal of this study was to explore the associations of perinatal exposure to the various gaseous pollutants (O_3_, SO_2_, NO_2_, and CO) with ASD risk. We additionally considered the influence of PM_2.5_ as we have previously found such exposure to be associated with ASD in this cohort (Raz et al. 2015). To do this we leveraged the Nurses’ Health Study II (NHS II), a large cohort of nurses across the continental U.S. in which ASD cases among the children of the nurses have been identified.

## Material and methods

### Population/outcome measurement

The study participants were the children of the NHS II participants, a prospective cohort of 116,430 U.S. female nurses recruited in 1989 who have been followed biennially by questionnaire mailed to their residential address (Solomon et al. 1997). NHS II participants were originally recruited from 14 U.S. states, but they now reside in all 50 states (Solomon et al. 1997). The study was approved by the Partners Health Care Institutional Review Board and Harvard T.H. Chan School of Public Health complied with all applicable U.S. regulations; the return of completed questionnaires constituted consent to participate.

In 2005, the nurses were asked if any of their children had been diagnosed with autism, Asperger’s syndrome, or other ASD listed as separate responses. In 2007–2008, we sent a supplemental questionnaire to the 756 women who had reported having a child with any of these conditions, and 636 mothers (84%) responded. The follow-up questionnaire included the child’s sex, birth date, and whether they were adopted. Also, if a mother reported having more than one child with ASD, she was directed to report the youngest child. Controls were randomly selected from the full cohort, and frequency matched at a 1:4 ratio by the years in which case mothers reported births and reported not having a child with ASD on the 2005 questionnaire, to yield a total of 3,000 controls who were also sent a supplemental questionnaire. Of these, 2,747 (92%) responded.

Because the nurses’ addresses before the start of NHS II in June 1989 were unknown, we only included the 265 ASD cases with an estimated conception month at this time or later. We excluded 2 of the cases with genetic syndromes associated with ASD (2 Rett syndrome) and 4 for whom ASD was not confirmed by the mother on the follow-up questionnaire. We further excluded 9 controls who were reported to have ASD on the 2009 questionnaire and children with missing birth months (7 cases and 80 controls). We also excluded children whose addresses could not be geocoded (4 cases and 12 controls). The final study sample consisted of 250 cases and 1539 controls with any pollutant data, although numbers for any given pollutant analysis varied based on available pollutant data. None of these children were reported to have been adopted.

The ASD diagnosis was validated by telephone administration of the Autism Diagnostic Interview-Revised (ADI-R) (Lord et al. 1994) in a subsample of 50 randomly selected cases from mothers who indicated willingness to complete the interview (81% of the 636 mothers). 43 children (86%) met full ADI-R criteria for autistic disorder (based on minimum scores in all three domains and onset by 3 years of age). Others met the onset criterion and communication domain score (n = 5) or met the cutoff in one or two domains only (n = 2). Thus, all children in the subsample exhibited some autistic behaviors.

### Exposure

The residential location of the nurses was determined from the mailing addresses used for the biennial NHSII questionnaire at the approximate time of the index child’s birth. It was geocoded and classified according to individual’s residential latitude and longitude. Children born through 1990 were assigned the geographic location of their mother in 1989 (the first year of study). Children born in 1991 or 1992 were assigned the mother’s mailing address in 1991, and births from 1993 through 2002 were assigned the mother’s addresses, updated every other year, in a similar manner.

The monthly average for each gaseous pollutant O_3_ (ppm), NO_2_ (ppb), SO_2_ (ppb), and CO (ppm) was obtained from daily values measured at the nearest 5 monitoring sites weighted by inverse of the squared distance between individual’s residential latitude and longitude and monitoring site. Data were obtained from EPA’s Air Quality System (AQS) database. Monthly ambient predictions of PM_2.5_ were generated from a nationwide spatiotemporal model (Yanosky et al. 2009a, b) that used the monthly average PM_2.5_ values from the US EPA’s Air Quality System. The models incorporated various geospatial predictors, including distance to roads, population density, point sources (such as power-generating utilities and waste combustors), elevation, and meteorological factors using generalized additive models (Yanosky et al. 2008). Due to limited PM_2.5_ monitoring data before 1999, PM_2.5_ levels for this period were estimated using PM_10_ and visibility data from airports (Yanosky et al. 2009a, b). These models produce estimates for any location in the conterminous U.S. on a monthly basis. They have been shown to exhibit low bias and high precision, with a normalized mean bias of –1.6% and an absolute prediction error of 1.61 for PM_2.5_.

We estimated the exposure to air pollutants before, during, and after pregnancy for each child, averaging monthly concentrations for the mother’s residential address during pregnancy and the 9 months before and after, or three-month intervals corresponding with the trimesters of pregnancy and three months before and after pregnancy. The months of pregnancy were determined from the child’s birth month and gestational age at birth reported by the mother.

### Statistical analysis

**Fig. 1:**
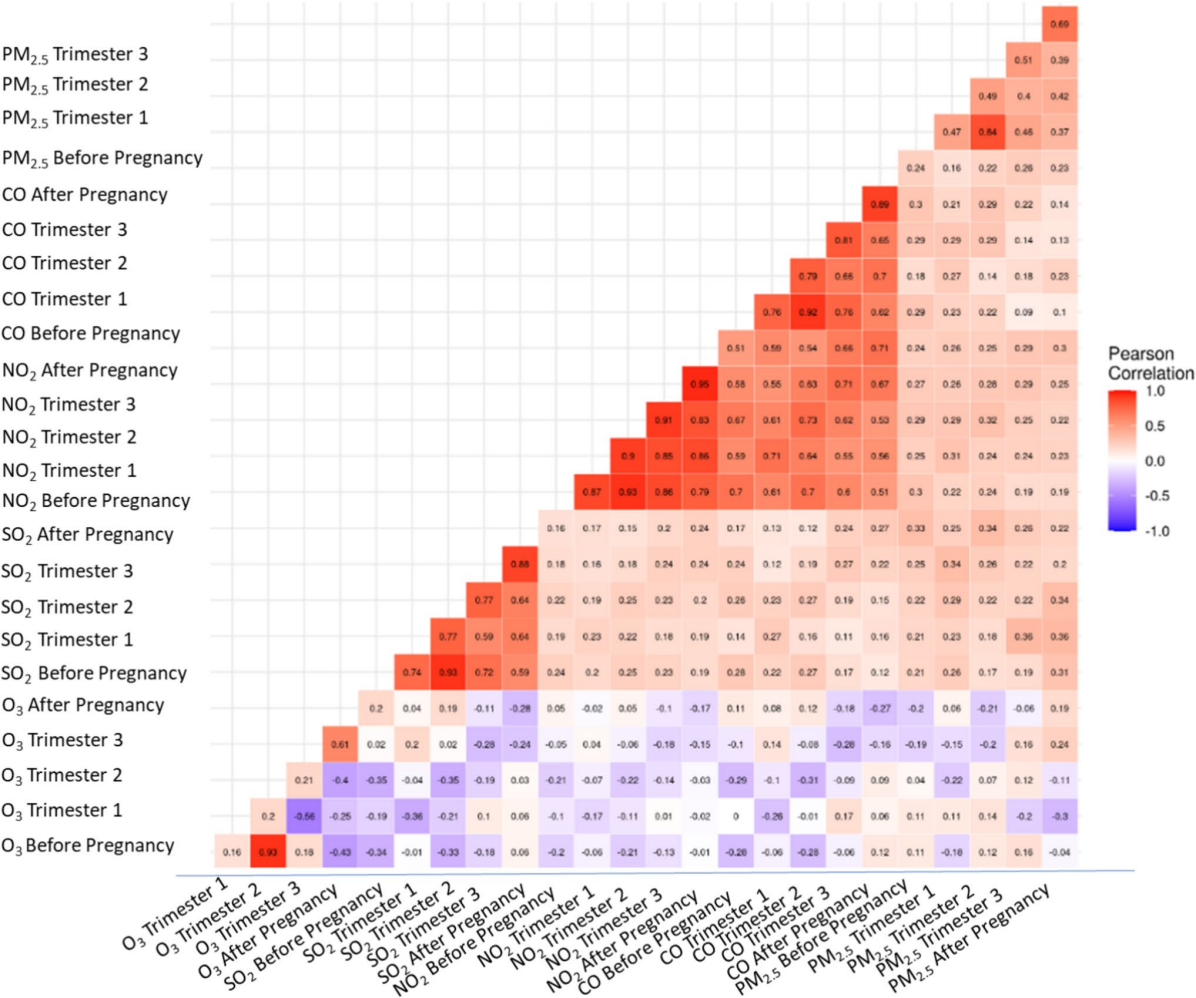
Correlation matrix showing the Pearson correlation coefficients of the air pollutant values from the different 3-month exposure periods: 3 months preconception; first, second, and third trimester; and first 3 months after birth. Pearson correlation coefficients range from −1 to 1, with values closer to −1 indicating higher negative correlation, values closer to 0 indicating no correlation, and values closer to 1 indicating higher positive correlation

Logistic regression models were used to estimate the odds ratio (OR) and 95% confidence interval (CI) for ASD associated with specific air pollutant exposures during different exposure windows around pregnancy. We examined the correlations between the different exposures in different time periods and among different pollutants. Correlations across different exposure time periods for the same pollutant were much higher than across pollutants other than for NO_2_ and CO (Fig. 1 & Figure S1). Because co-adjustment for all pollutants and all exposure windows would lead to very unstable models, we analyzed each pollutant separately but considered multiple exposure periods for a given pollutant simultaneously in one model (mutually adjusted model) to account for co-exposure confounding by the same pollutant at different exposure windows. For example, when analyzing CO a mutually adjusted model would include the CO exposure values for the 9 months before conception, the whole pregnancy, and the 9 months after birth all within the same statistical model.

We adjusted for year of birth (continuous), birth month (categorical), child sex (categorical), maternal age at birth (continuous), paternal/partner’s education (high school or less, some college, college diploma or higher), median census tract income (continuous), and census tract population density (continuous). All covariate data except for census variables were from maternal self-report. Among these covariates, 6 cases and 40 controls for the census tract income and 11 cases 179 controls for the paternal age had missing data. We used the missing indicator method for missing data for the covariates. All covariate data except for census variables were from maternal self-reports.

### Sensitivity analysis

Sensitivity analyses adjusting gaseous pollutant models for PM_2.5_ were also conducted because of the contribution of some of the pollutants to PM_2.5_. Also, additional analyses were done exclusively in males since the number of female cases was small (n = 39). Finally, we conducted analyses restricting the study population to the children of NHSII participants who did not change residence (non-movers) between pre- and post-pregnancy, as the exact moving date was unknown (165 cases and 1001 controls).

We used SAS version 9.3 (SAS Institute Inc., Cary, NC) for data extraction and R version 4.2.0 (http://www.r-project.org/foundation/) for Linux-gnu for analyses.

## Results

### Participant characteristics

Our nested-case control study identified 250 ASD cases and 1,539 controls. ASD cases were more likely to be male (cases: 84%, controls: 52%) and have mothers who smoked during pregnancy (cases: 9%, controls: 3%) (Table 1). As expected from having matched by year, the median year of birth was 1993, and race/ethnicity, maternal age at birth, paternal age at birth, and birthweight were also comparable across groups.

**Table 1:** Study population characteristics by ASD status, Nurses’ Health Study II

Characteristics	Cases (250)	Controls (1539)
Male sex [*n (%)*]	211 (84)	799 (52)
Year of birth [*median (IQR)*]	1993 (5)	1993 (5)
Maternal age at birth [*median (IQR)*]	34.0 (4.0)	33.6 (3.7)
Paternal age at birth [*median (IQR)*]	36.9 (5.2)	36.3 (4.6)
Median census income ($1,000) [*median (IQR)*]	64 (20)	65 (22)
Median census house value ($1,000) [*median (IQR)*]	177 (119)	168 (113)
Birth weight [*mean (sd)*]	7.1 (1.5)	7.2 (1.3)
Father’s education [*n (%)*]		
High school	34 (14)	210 (14)
2-year college	43 (17)	210 (14)
4-year college	80 (32)	548 (36)
Graduate school	75 (30)	502 (33)
Missing	15 (6)	59 (4)
Parent’s Marital Status [*n (%)*]		
Married	190 (76)	1175 (76)
Never married	51 (20)	270 (18)
Other	9 (4)	94 (6)
Mother Smoking during pregnancy [*n (%)*]		
Yes	22 (9)	50 (3)
No	163 (65)	1112 (72)
Missing	65 (26)	377 (24)

### Air pollutants exposure

The air pollution exposure range before, during, and after pregnancy was: 0.005 ppm to 0.06 ppm for O_3_, 0 ppb to 25.45 ppb for SO_2_, 0.42 ppb to 50.94 ppb for NO_2_, 0.06 ppm to 2.44 ppm for CO. The median values were similar before, during, and after pregnancy for each air pollutant (Table S1).

### Association between Air Pollutants and Odds of ASD

#### 0_3_

When looking at exposure overall pregnancy and the 9 months before and after pregnancy (Table 2), there were no notable associations with ASD, although the OR was slightly elevated during pregnancy in the mutually adjusted model. In the three-month exposure models (Table 2), there was a slight, but not significant, elevation in OR for exposure in the second trimester in separate models that became more pronounced, although still not reaching statistical significance, in the mutually adjusted model (OR per 0.007 ppm [interquartile range; IQR]: 1.20; 95% CI: 0.96–1.50). In the adjusted model, there was an elevated OR in the 1 st trimester of pregnancy (OR per IQR = 1.13; 95% CI: 1.01–1.26).

**Table 2:** Odds ratios (OR) and 95% confidence intervals (CI) for ASD per interquartile range (IQR) increase in air pollutants in different exposure windows

		O_3_ (N = 1782; cases = 250; controls = 1532)		SO_2_ (N = 1770; cases = 250; controls = 1520)		NO_2_ (N = 1731; cases = 244; controls = 1487)		CO (N = 1765; cases = 249; controls = 1516)	
Model		Adjusted^*^	Mutually adjusted^**^	Adjusted^*^	Mutually adjusted^**^	Adjusted^*^	Mutually adjusted^**^	Adjusted^*^	Mutually adjusted^**^
Before conception	9 months	0.80 (0.66, 0.96)	0.73 (0.58, 0.90)	1.19 (0.97, 1.46)	2.26 (1.33, 3.83)	1.32 (1.13, 1.54)	1.02 (0.62, 1.69)	1.31 (1.08, 1.59)	1.08 (0.71, 1.66)
	3 months	0.93 (0.81, 1.06)	0.97 (0.73, 1.29)	1.19 (0.99, 1.44)	1.63 (0.97, 2.74)	1.27 (1.09, 1.47)	0.90 (0.53, 1.54)	1.20 (1.01, 1.43)	0.96 (0.61, 1.51)
During pregnancy	whole pregnancy	1.01 (0.82, 1.25)	1.13 (0.85, 1.50)	1.05 (0.85, 1.29)	0.51 (0.25, 1.03)	1.34 (1.15, 1.57)	1.62 (0.86, 3.05)	1.32 (1.09, 1.61)	1.13 (0.66, 1.92)
	Trimester 1	1.13 (1.01, 1.26)	0.98 (0.79, 1.21)	1.02 (0.85, 1.22)	0.74 (0.50, 1.10)	1.24 (1.07, 1.44)	0.95 (0.56, 1.63)	1.14 (0.96, 1.35)	0.80 (0.54, 1.20)
	Trimester 2	1.07 (0.94, 1.23)	1.20 (0.96, 1.50)	1.04 (0.86, 1.25)	1.14 (0.75, 1.74)	1.31 (1.12, 1.52)	0.93 (0.53, 1.62)	1.28 (1.08, 1.51)	1.30 (0.86, 1.95)
	Trimester 3	0.85 (0.76, 0.96)	0.75 (0.55, 1.02)	1.07 (0.88, 1.30)	0.75 (0.40, 1.38)	1.38 (1.17, 1.61)	2.10 (1.03, 4.28)	1.35 (1.12, 1.61)	0.98 (0.53, 1.79)
After birth	9 months	1.06 (0.86, 1.30)	1.12 (0.87, 1.46)	1.08 (0.86, 1.35)	0.95 (0.48, 1.87)	1.32 (1.12, 1.56)	0.80 (0.44, 1.45)	1.34 (1.09, 1.65)	1.11 (0.66, 1.86)
	3 months	0.87 (0.77, 0.98)	1.18 (0.83, 1.67)	1.15 (0.94, 1.39)	1.05 (0.51, 2.18)	1.33 (1.13, 1.56)	0.80 (0.41, 1.57)	1.34 (1.11, 1.62)	1.36 (0.71, 2.63)

^*^ORs are adjusted for child sex, year of birth, month of birth, maternal age at birth, paternal age at birth, and census income

^**^Mutually adjusted for all exposure periods for the same air pollutant, as well as all other covariates listed above

IQR: O_3_ (0.007 ppm); SO_2_ (5.54 ppb); NO_2_ (9.86 ppb); CO (0.52 ppm);

#### SO_2_

When looking at exposure overall pregnancy and the 9 months before and after pregnancy (Table 2), there was slightly elevated odds of ASD in the 9 months prior to pregnancy that became more pronounced in the mutually adjusted model (OR per 5.54 ppb [IQR]: 2.26; 95% CI: 1.33–3.83). These results were similar, although slightly weaker when considering the three-month exposure windows (Table 2). Also, when the three trimesters of pregnancy were included in a model with the 9 months before and after pregnancy, the OR for ASD was significantly elevated for exposure before pregnancy (OR per IQR: 2.92; 95% CI: 1.60–5.33; Table S2).

#### NO_2_

When looking at exposure overall pregnancy and the 9 months before and after pregnancy (Table 2), there were similarly significantly elevated OR for each exposure period. In the mutually adjusted model, the only period that remained elevated was the pregnancy period, in which the OR for ASD per 9.86 ppb (IQR) was 1.34 (95% CI: 1.15–1.57) in a model for that period only, and 1.62 (95% CI: 0.86–3.05) in the mutually adjusted model. When looking at three-month exposure windows (Table 2), there was also a similar significantly elevated OR for all exposure periods when analyzed separately, while in the mutually adjusted model, the only elevated OR was in the 3rd trimester of pregnancy (OR per IQR = 3.10; 95% CI: 1.03–4.28; in the separate model the 3rd trimester OR = 1.38; 95% CI: 1.17–1.61).

#### CO

When looking at exposure over all pregnancy and the 9 months before and after pregnancy (Table 2), there were similarly significantly elevated OR for each exposure period. In the mutually adjusted model, the most elevated period was the post-pregnancy period, in which the OR for ASD per 0.52 ppm (IQR) was 1.34 (95% CI: 1.09–1.65) in a model for that period only, and 1.11 (95% CI: 0.66–1.86) in the mutually adjusted model. When looking at three-month exposure windows (Table 2), there was also similar significantly elevated OR for all exposure periods when analyzed separately, while in the mutually adjusted model no period was significant, but both trimester 2 and the three months post-pregnancy were elevated. However, when the three trimesters of pregnancy were included in a model with the 9 months before and after pregnancy, the OR for ASD was elevated for exposure in the 9 months after birth (OR per IQR: 1.49; 95% CI: 0.81–2.75; Table S2).

### Sensitivity analysis

When adjusting for PM_2.5_ (Tables S3), results were generally similar, except that the OR for NO_2_ during all pregnancy and 3rd trimester were notably weaker, and results for CO after pregnancy became stronger. When considering sex differences, the male population results were similar to those in the whole population, although the association with CO by 3-month periods appeared more in the 2nd trimester rather than the 3 months after birth (Table S4 and Table S5). The female population has shown different association for O_3_ and SO_2_ compared to the male and whole population; higher risk for O_3_ 9 months before conception (OR for female population: 1.04; 95% CI: 0.64, 1.71, OR for male population: 0.67; 95% CI: 0.53, 0.86 in mutually adjusted model) and lower association for SO_2_ (OR for female population: 0.79; 95% CI: 0.17, 3.72, OR for male population: 2.47; 95% CI: 1.36, 4.47 in mutually adjusted model). When restricting the population to non-movers (mothers for whom the pre- and post-pregnancy addresses were identical) (Table S6), the associations were similar with most key findings slightly stronger other than for O_3_ in the 2nd trimester, which was slightly weaker (OR: 1.23; 95% CI: 0.95, 1.58).

## Discussion

In this nested case–control study across the continental U.S., we found that exposure to each of the gaseous criteria air pollutants O_3_, SO_2_, NO_2_, and CO was associated with increased odds of having a child diagnosed with ASD, but in different periods. In general, associations appeared more robust with shorter time windows, with O_3_ in the 2nd trimester, NO_2_ in the 3rd trimester, and CO in the 2nd trimester or 3 months after pregnancy as we have reported (Raz et al. 2015). However, the association with SO_2_ appeared strongest for the 9 months before pregnancy, and similarly, the association with CO seemed somewhat stronger for the 9 months after pregnancy. Our study results highlight the importance of mutual adjustment for different correlated exposure periods to identify critical windows of exposure; in non-adjusted models, several periods appeared to be associated with ASD, and in some cases no period was. Such time-period co-adjustment removes co-exposure confounding bias (in this case by the same pollutant in a different time window) (Schisterman et al. 2017). Mutually-adjusted models, however, will be more unstable because of the correlated variables (Schisterman et al. 2017), so if only one critical window of exposure is identified in mutually-adjusted models, it may be better to interpret the results from the single window model, for example, our results for NO_2_ and CO. Of note, though, mutual adjustment for other exposure windows can help minimize confounding by factors associated with exposures in the different time periods, even unknown ones because adjusting for an effect of a variable partially adjusts for the variable itself (Greenland 1980, Ogburn and Vanderweele 2013).

### Ozone

We observed a significantly increased odds ratio for O_3_ during the 2nd trimester of pregnancy. A study in Los Angeles, USA reported an association between O_3_ exposure during pregnancy and a 6% increased odds of ASD per 11.54 ppb increase in O_3_ (Becerra et al. 2013). This finding was slightly stronger when co-adjusted for NO, NO_2_, or PM_2.5_, and there was the suggestion of a stronger effect in the 3rd trimester, although mutual adjustment for different exposure windows was not done. Another study in California found no association between O_3_ and ASD, either considering the whole pregnancy or pregnancy trimesters, although mutual adjustment for different exposure periods was not done (Volk et al. 2013), scenarios in which we also did not see associations. In Taiwan, a 59% increase in the hazard ratio per 100 ppb increase in early childhood ozone exposure was found (Jung et al. 2013), and a study in Ohio detected an elevated OR for the 2nd year after the pregnancy (Kaufman et al. 2019). A Korean study found a paradoxical reduction in the odds of ASD with O_3_ exposure during pregnancy (Lee et al. 2023), which possibly could relate to the usually negative correlation between O_3_ and other air pollutants.

### SO_2_

We observed an increased odds ratio of ASD for SO_2_ exposures in the 9 months before pregnancy that was particularly pronounced and significant when mutually adjusting for pregnancy trimesters and the 9 months after pregnancy. Few studies have explored SO_2_ and ASD (Chun et al. 2020). One study in Korea found that the incidence of ASD increased when infants were exposed to SO_2_ during pregnancy, especially in the 3rd trimester with an OR of 2.72 (Lee et al. 2023). However, this study did not mutually adjust for different time windows of exposure or consider exposures before or after pregnancy. A study in Denmark found an OR of 1.21 per IQR increase in SO_2_ in the 9 months after pregnancy (Ritz et al. 2018), that persisted when adjusting for both the pre-pregnancy and pregnancy periods, but results for the pre-pregnancy period were not shown. A study in Iran found no association between SO_2_ and ASD (Yousefian et al. 2018).

### NO_2_

Our finding of increased odds of ASD with higher exposure to NO_2_ during pregnancy—especially in the 3rd trimester—is generally consistent with previous findings, with associations frequently seen during pregnancy and often later in pregnancy (Flores-Pajot et al. 2016), or even after pregnancy (Goodrich et al. 2018; Ritz et al. 2018). However, some studies found no association between NO_2_ during pregnancy and autism traits or ASD (Guxens et al. 2016; Pagalan et al. 2019). These studies, however, did not look at periods at finer time resolution than all pregnancies, which could have contributed to null findings, nor did they co-adjust for other exposure periods. Some of these also did not have clinical ASD as the outcome but rather used behavioral scales for the outcome assessment. Our finding of a specific critical window for NO₂ in the third trimester is consistent with the recent large-scale data from the ECHO Consortium, which also identified significant associations between prenatal air pollution and ASD across diverse U.S. populations (Ghassabian et al. 2025). This alignment suggests that the neurodevelopmental impact of NO₂ may be consistent even across geographically and demographically varied cohorts.

In our study results, when mutually adjusting NO_2_ exposures for PM_2.5_, the association with NO_2_ became weaker. This could suggest that the NO_2_ association is confounded by other aspects of PM, but it must also be considered that PM_2.5_ and NO_2_ exposures are correlated and our PM_2.5_ exposure data (spatiotemporal model) is at higher spatial resolution compared to the NO_2_ data (monitoring station-based). Thus, our PM_2.5_ measures may actually capture true NO_2_ exposures better than the nearest monitor average does, in which case the PM_2.5_ measure would effectively take on any NO_2_ effect.

### CO

We observed an increased odds ratio of ASD for CO exposures after pregnancy that was particularly pronounced when adjusted for PM_2.5_ or when considering exposure in the 9 months after pregnancy and mutually adjusting for pregnancy trimesters. Only a few prior studies have examined CO and ASD. One study in Iran found a significant association between CO exposure and autism in the 2nd trimester (1 ppm increase in CO resulted in 59% increase in OR) (Ghahari et al. 2023). Another in Taiwan found CO exposures in all three trimesters were associated with increased risk of developing ASD (1 ppm increase in CO was associated with HR of 1.93, 1.77, and 1.75 for 1 st, 2nd, and 3rd trimester respectively) (Wang et al. 2021). However, neither of these mutually adjusted for different exposure periods or considered periods before or after pregnancy. A study in Los Angeles found no association with CO during pregnancy (Becerra et al. 2013).

### Mechanisms

There are various pathways to explain the association between exposure to air pollution and the onset of ASD. Exposure to various air pollutants can induce neuroinflammation, microglial activation, and oxidative stress (Hine et al. 1970; Meng 2003; Pereyra-Muñoz et al. 2006; Guevara-Guzmán et al. 2009; Persico and Merelli 2014), which have been found to be associated with ASD in children (Frustaci et al. 2012; Cheng et al. 2016). Microglia play a role in synaptic pruning, which occurs from the last trimester of pregnancy through the early years of life, alterations in which could lead to altered brain connectivity patterns found in children with ASD (Schafer and Stevens 2015; Mohammad-Rezazadeh et al. 2016; Roqué et al. 2016). This could be linked to our study results with high odds of ASD with exposure to air pollutants in the later stage of pregnancy or after birth (O_3_: 2nd trimester, NO_2_: 3rd trimester, and CO: 3 or 9 months after birth). O_3_ may induce oxidative stress and inflammation, which can have a significant impact during sensitive periods of brain development. The 2nd trimester is a period of tremendous neurodevelopment, including neurogenesis, differentiation, migration, and synaptogenesis (Costa et al. 2019). Later in pregnancy (e.g., the 3rd trimester) many of these processes are continuing and more refinement of neuronal connectivity is occurring that could be disrupted by NO_2_ (Morrel et al. 2024). Post-birth additional developmental processes start to ramp up, such as synaptic pruning and myelination, which could potentially be disrupted by impairment of oxygen delivery by CO (Costa et al. 2019). Our finding of exposure to SO_2_ 9 months before pregnancy and increased odds of ASD is intriguing and raises the issue of whether paternal effects may be relevant, although pre-pregnancy effects on ova are also possible. Recent research has suggested that epigenetic changes in sperm are associated with child quantitative autistic traits, highlighting both the paternal role and the preconception exposures’ contribution to risks (Cescon et al. 2020; Feinberg et al. 2024).

A critical consequence of air pollution-induced dysbiosis and respiratory inflammation is maternal immune activation (MIA), a well-recognized risk factor for ASD (Ayoub 2025). Inhalation of gaseous pollutants can trigger a systemic inflammatory response in the mother, characterized by the elevation of pro-inflammatory cytokines (Vadillo-Ortega et al. 2014), which could directly interfere with fetal neurodevelopmental processes. Maternal exposure to gaseous pollutants may shift the fetal immune environment toward a pro-inflammatory state, and increase the offspring's susceptibility to the neurological phenotypes associated with ASD. This state of MIA may further serve as a catalyst for long-term neurodevelopmental effects through epigenetic modifications. Recent evidence suggests that prenatal exposure to ambient air pollution is associated with accelerated epigenetic aging at birth, indicating that such exposures can alter the molecular development of the newborn (Song et al. 2022). These epigenetic changes may serve as a critical link between early-life environmental exposure and the occurrence of ASD.

Also, emerging evidence suggests that the lung-gut axis may also play a significant role in linking air pollution to ASD risk (Zhang et al. 2025). Inhalation of gaseous pollutants can alter the composition of the lung microbiota, triggering a cascade of respiratory inflammation that disrupts homeostatic communication between the lungs and the gastrointestinal tract (Pang et al. 2025). This disruption can lead to intestinal dysbiosis, which is frequently observed in children with ASD, this may further exacerbate systemic inflammation and influence neurodevelopmental disorder (Zarimeidani et al. 2025). Integrating the role of the lung microbiota provides a more comprehensive framework for understanding how maternal respiratory exposures to gaseous pollutants might be associated with ASD.

Our study findings suggest different exposure periods to be most relevant for different pollutants. If true, this would seem to suggest that different mechanisms may be at play for the different pollutants rather than common mechanistic pathways like general neuroinflammation. This could have important implications for understanding the pathogenesis of ASD, but further confirmation of such differences in associations by exposure windows in much larger epidemiology studies combined with more refined exposure assessment and consideration of potential confounding of one exposure window by another are still needed. At the same time, laboratory experiments to determine whether different developmental processes are particularly sensitive to different gaseous pollutants, and possibly in specific windows of exposure, would also help inform this question.

### Limitations

As mentioned, the averaging of nearest monitors is a limitation of the study. This likely results in measurement error of the ambient exposure. Additionally, error in actual personal exposure is also present when using any ambient exposure level to predict personal exposure. However, the measurement error this cause is likely non-differential with respect to ASD, and so would likely bias any true results towards the null (Hart et al. 2015). On the other hand, the use of these ambient estimates of exposure protects from confounding by individual behavioral factors (Weisskopf and Webster 2017). Our findings do point to the importance of co-adjustment for exposure in other time periods (Weisskopf et al. 2015). The correlation between exposures to the same pollutant in other time periods was generally much higher than correlations with other air pollutants making the former more important to address. Nonetheless, residual confounding by other pollutants is still possible. Further, confounding by other pollutants seems more likely to be something that would occur in the same exposure time window, e.g. not accounting for a causal effect of NO_2_ in one exposure window would make the CO in the same window look associated with ASD. However, we generally found associations with different pollutants in different time windows, seemingly reducing the concern about co-exposure confounding. Additionally, our study findings could have residual or unmeasured confounding due to unaccounted-for factors, such as socioeconomic status, genetic predispositions, or other environmental exposures. However, to the extent that these would affect exposure in all time windows, mutually adjusting for other exposure windows helps control that confounding because adjusting for effects of factors partially adjusts for the factor (Greenland 1980, Ogburn and Vanderweele 2013). Further, when some exposure windows in mutually adjusted models do not show an association with the outcome, this suggests a lack of confounding by common causes of exposure in the different windows based on the negative control exposure principle (Lipsitch et al. 2010; Weisskopf et al. 2016). Furthermore, our study may be subject to live birth bias (Leung et al. 2021). Because we only included children who survived to an age where an ASD diagnosis could be made, we cannot account for the potential impact of gaseous pollutants on pregnancy loss. However, while live birth bias is a potential concern in prenatal studies, the presence of strong positive associations in later developmental windows (e.g., the third trimester and post-pregnancy) suggests that our findings are more likely driven by neurodevelopmental impacts than by differential fetal survival.

Our findings suggest that perinatal exposure to different air pollutants may affect the risks of ASD at specific periods during development: notably SO_2_ in the pre-pregnancy period, O_3_ in the second trimester, NO_2_ in the third trimester, and CO in the post-pregnancy period. More exploration of this with more refined exposure measurements and larger studies could help the understanding of these details, but attention to confounding by exposure in other time windows will be critical. Combined with mechanistic research into possible pollutant and timing-specific effects on neurodevelopmental processes could help further the understanding of ASD pathogenesis. While our study leverages the well-characterized NHS II cohort, consortium-wide analysis demonstrates the power of pooled data in confirming these associations. Such large-scale studies are essential to definitively isolate the effects of individual gaseous pollutants from the broader pollution mixture. Furthermore, to move beyond statistical associations, future research must integrate biological pathways into these large-scale frameworks. Given the emerging evidence linking the lung-gut axis to neurodevelopment, future studies should investigate the role of the host microbiome as a potential mediator of the association between air pollution and ASD.

## Supplementary Information

Below is the link to the electronic supplementary material.

Supplementary file1 (DOCX 411 kb)

## Data Availability

The data that support the findings of this study are available on request from the corresponding author. The data are not publicly available due to privacy or ethical restrictions.

## References

[CR1] American Psychiatric Association, D. and A. P. Association (2013). Diagnostic and statistical manual of mental disorders: DSM-5, American psychiatric association Washington, DC.

[CR2] Ayoub G (2025) Neurodevelopmental impact of maternal immune activation and autoimmune disorders, environmental toxicants and folate metabolism on autism spectrum disorder. Curr Issues Mol Biol 47(9):721 41020843 10.3390/cimb47090721 PMC12469020

[CR3] Becerra TA, Wilhelm M, Olsen J, Cockburn M, Ritz B (2013) Ambient air pollution and autism in Los Angeles county, California. Environ Health Perspect 121(3):380–386 23249813 10.1289/ehp.1205827 PMC3621187

[CR4] Cescon M, Chianese R, Tavares RS (2020) Environmental impact on male (in)fertility via epigenetic route. J Clin Med. 10.3390/jcm9082520 10.3390/jcm9082520 PMC7463911 32764255

[CR5] Cheng H, Saffari A, Sioutas C, Forman HJ, Morgan TE, Finch CE (2016) Nanoscale particulate matter from urban traffic rapidly induces oxidative stress and inflammation in olfactory epithelium with concomitant effects on brain. Environ Health Perspect 124(10):1537–1546 27187980 10.1289/EHP134 PMC5047762

[CR6] Chun H, Leung C, Wen SW, McDonald J, Shin HH (2020) Maternal exposure to air pollution and risk of autism in children: a systematic review and meta-analysis. Environ Pollut 256:113307 31733973 10.1016/j.envpol.2019.113307

[CR7] Costa LG, Cole TB, Dao K, Chang YC, Garrick JM (2019) Developmental impact of air pollution on brain function. Neurochem Int 131:104580 31626830 10.1016/j.neuint.2019.104580 PMC6892600

[CR8] Crump KS, Kjellström T, Shipp AM, Silvers A, Stewart A (1998) Influence of prenatal mercury exposure upon scholastic and psychological test performance: benchmark analysis of a New Zealand cohort. Risk Anal 18:701–713 9972579 10.1023/b:rian.0000005917.52151.e6

[CR9] Dutheil F, Comptour A, Morlon R, Mermillod M, Pereira B, Baker JS, Charkhabi M, Clinchamps M, Bourdel N (2021) Autism spectrum disorder and air pollution: a systematic review and meta-analysis. Environ Pollut 278:116856 33714060 10.1016/j.envpol.2021.116856

[CR10] Feinberg JI, Schrott R, Ladd-Acosta C, Newschaffer CJ, Hertz-Picciotto I, Croen LA, Fallin MD, Feinberg AP, Volk HE (2024) Epigenetic changes in sperm are associated with paternal and child quantitative autistic traits in an autism-enriched cohort. Mol Psychiatry 29(1):43–53 37100868 10.1038/s41380-023-02046-7 PMC12450096

[CR11] Flores-Pajot MC, Ofner M, Do MT, Lavigne E, Villeneuve PJ (2016) Childhood autism spectrum disorders and exposure to nitrogen dioxide, and particulate matter air pollution: a review and meta-analysis. Environ Res 151:763–776 27609410 10.1016/j.envres.2016.07.030

[CR12] Frustaci A, Neri M, Cesario A, Adams JB, Domenici E, Dalla Bernardina B, Bonassi S (2012) Oxidative stress-related biomarkers in autism: systematic review and meta-analyses. Free Radic Biol Med 52(10):2128–2141 22542447 10.1016/j.freeradbiomed.2012.03.011

[CR13] Ghahari N, Yousefian F, Najafi E (2023) Prenatal exposure to ambient air pollution and autism spectrum disorders: results from a family-based case-control study. JCPP Adv 3(1):e12129 37431319 10.1002/jcv2.12129 PMC10241453

[CR14] Ghassabian A, Dickerson AS, Wang Y, Braun JM, Bennett DH, Croen LA, LeWinn KZ, Burris HH, Habre R, Lyall K, Frazier JA, Glass HC, Hooper SR, Joseph RM, Karr CJ, Schmidt RJ, Friedman C, Karagas MR, Stroustrup A, Straughen JK, Dunlop AL, Ganiban JM, Leve LD, Wright RJ, McEvoy CT, Hipwell AE, Giardino AP, Santos HP, Jr., Krause H, Oken E, Camargo CA, Jr., Oh J, Loftus C,. O'Shea TM,. O'Connor TG, Szpiro A and Volk HE (2025). "Prenatal Air Pollution Exposure and Autism Spectrum Disorder in the ECHO Consortium." Environ Health Perspect. 10.1021/EHP.6c00106 PMC13347653 42428257

[CR15] Glasson EJ, Bower C, Petterson B, de Klerk N, Chaney G, Hallmayer JF (2004) Perinatal factors and the development of autism: a population study. Arch Gen Psychiatry 61(6):618–627 15184241 10.1001/archpsyc.61.6.618

[CR16] Goodrich AJ, Volk HE, Tancredi DJ, McConnell R, Lurmann FW, Hansen RL, Schmidt RJ (2018) Joint effects of prenatal air pollutant exposure and maternal folic acid supplementation on risk of autism spectrum disorder. Autism Res 11(1):69–80 29120534 10.1002/aur.1885 PMC5777535

[CR17] Grandjean P, Landrigan PJ (2006) Developmental neurotoxicity of industrial chemicals. Lancet 368(9553):2167–2178 17174709 10.1016/S0140-6736(06)69665-7

[CR18] Greenland S (1980) The effect of misclassification in the presence of covariates. Am J Epidemiol 112(4):564–569 7424903 10.1093/oxfordjournals.aje.a113025

[CR19] Guevara-Guzmán R, Arriaga V, Kendrick K, Bernal C, Vega X, Mercado-Gómez O, Rivas-Arancibia S (2009) Estradiol prevents ozone-induced increases in brain lipid peroxidation and impaired social recognition memory in female rats. Neuroscience 159(3):940–950 19356678 10.1016/j.neuroscience.2009.01.047

[CR20] Guxens M, Ghassabian A, Gong T, Garcia-Esteban R, Porta D, Giorgis-Allemand L, Almqvist C, Aranbarri A, Beelen R, Badaloni C (2016) Air pollution exposure during pregnancy and childhood autistic traits in four European population-based cohort studies: the ESCAPE project. Environ Health Perspect 124(1):133–140 26068947 10.1289/ehp.1408483 PMC4710593

[CR21] Hallmayer J, Cleveland S, Torres A, Phillips J, Cohen B, Torigoe T, Miller J, Fedele A, Collins J, Smith K (2011) Genetic heritability and shared environmental factors among twin pairs with autism. Arch Gen Psychiatry 68(11):1095–1102 21727249 10.1001/archgenpsychiatry.2011.76 PMC4440679

[CR22] Hart JE, Liao X, Hong B, Puett RC, Yanosky JD, Suh H, Kioumourtzoglou MA, Spiegelman D, Laden F (2015) The association of long-term exposure to PM2.5 on all-cause mortality in the Nurses’ Health Study and the impact of measurement-error correction. Environ Health 14:38 25926123 10.1186/s12940-015-0027-6 PMC4427963

[CR23] Hertz-Picciotto I, Herr CE, Yap P-S, Dostál M, Shumway RH, Ashwood P, Lipsett M, Joad JP, Pinkerton KE, Šrám RJ (2005) Air pollution and lymphocyte phenotype proportions in cord blood. Environ Health Perspect 113(10):1391–1398 16203253 10.1289/ehp.7610 PMC1281286

[CR24] Hine C, Meyers F, Wright R (1970) Pulmonary changes in animals exposed to nitrogen dioxide, effects of acute exposures. Toxicol Appl Pharmacol 16(1):201–213 5416750 10.1016/0041-008x(70)90177-8

[CR25] Jung C-R, Lin Y-T, Hwang B-F (2013) Air pollution and newly diagnostic autism spectrum disorders: a population-based cohort study in Taiwan. PLoS ONE 8(9):e75510 24086549 10.1371/journal.pone.0075510 PMC3783370

[CR26] Kaufman JA, Wright JM, Rice G, Connolly N, Bowers K, Anixt J (2019) Ambient ozone and fine particulate matter exposures and autism spectrum disorder in metropolitan Cincinnati, Ohio. Environ Res 171:218–227 30684889 10.1016/j.envres.2019.01.013 PMC7232936

[CR27] Lee KS, Min WK, Choi YJ, Jin S, Park KH, Kim S (2023) The effect of maternal exposure to air pollutants and heavy metals during pregnancy on the risk of neurological disorders using the national health insurance claims data of South Korea. Medicina 59(5):951 37241184 10.3390/medicina59050951 PMC10222370

[CR28] Leung M, Kioumourtzoglou MA, Raz R, Weisskopf MG (2021) Bias due to selection on live births in studies of environmental exposures during pregnancy: a simulation study. Environ Health Perspect 129(4):47001 33793300 10.1289/EHP7961 PMC8043129

[CR29] Lin Y-C, Li Y-C, Shangdiar S, Chou F-C, Sheu Y-T, Cheng P-C (2019) Assessment of PM2. 5 and PAH content in PM2. 5 emitted from mobile source gasoline-fueled vehicles in concomitant with the vehicle model and mileages. Chemosphere 226:502–508 30953895 10.1016/j.chemosphere.2019.03.137

[CR30] Lipsitch M, Tchetgen Tchetgen E, Cohen T (2010) Negative controls: a tool for detecting confounding and bias in observational studies. Epidemiology 21(3):383–388 20335814 10.1097/EDE.0b013e3181d61eeb PMC3053408

[CR31] Lord C, Rutter M, Le Couteur A (1994) Autism diagnostic interview-revised: a revised version of a diagnostic interview for caregivers of individuals with possible pervasive developmental disorders. J Autism Dev Disord 24(5):659–685 7814313 10.1007/BF02172145

[CR32] Lyall K, Schmidt RJ, Hertz-Picciotto I (2014) Maternal lifestyle and environmental risk factors for autism spectrum disorders. Int J Epidemiol 43(2):443–464 24518932 10.1093/ije/dyt282 PMC3997376

[CR33] Meng Z (2003) Oxidative damage of sulfur dioxide on various organs of mice: sulfur dioxide is a systemic oxidative damage agent. Inhal Toxicol 15(2):181–195 12528046 10.1080/08958370304476

[CR34] Mohammad-Rezazadeh I, Frohlich J, Loo SK, Jeste SS (2016) Brain connectivity in autism spectrum disorder. Curr Opin Neurol 29(2):137–147 26910484 10.1097/WCO.0000000000000301 PMC4843767

[CR35] MohanKumar SM, Campbell A, Block M, Veronesi B (2008) Particulate matter, oxidative stress and neurotoxicity. Neurotoxicology 29(3):479–488 18289684 10.1016/j.neuro.2007.12.004

[CR36] Morrel J, Dong M, Rosario MA, Cotter DL, Bottenhorn KL, Herting MM (2024) A Systematic Review of Air Pollution Exposure and Brain Structure and Function during Development." medRxiv. Ogburn, E. L. and T. J. Vanderweele (2013). "Bias attenuation results for nondifferentially mismeasured ordinal and coarsened confounders. Biometrika 100(1):241–248 10.1093/biomet/ass054 PMC3761876 24014285

[CR37] Pagalan L, Bickford C, Weikum W, Lanphear B, Brauer M, Lanphear N, Hanley GE, Oberlander TF, Winters M (2019) Association of prenatal exposure to air pollution with autism spectrum disorder. JAMA Pediatr 173(1):86–92 30452514 10.1001/jamapediatrics.2018.3101 PMC6583438

[CR38] Pang X, Huang P, Huang S, Liu X (2025) The gut-lung axis: a new perspective on the impact of atmospheric particulate matter exposure on chronic obstructive pulmonary disease. Front Immunol 16:1657675 41262252 10.3389/fimmu.2025.1657675 PMC12623317

[CR39] Pereyra-Muñoz N, Rugerio-Vargas C, Angoa-Pérez M, Borgonio-Pérez G, Rivas-Arancibia S (2006) Oxidative damage in substantia nigra and striatum of rats chronically exposed to ozone. J Chem Neuroanat 31(2):114–123 16236481 10.1016/j.jchemneu.2005.09.006

[CR40] Persico AM, Merelli S (2014) Environmental factors in the onset of autism spectrum disorder. Curr Dev Disord Rep 1:8–19

[CR41] Prevention, C. f. D. C. a. Autism spectrum disorder (ASD): data & statistics.

[CR42] Raz R, Roberts AL, Lyall K, Hart JE, Just AC, Laden F, Weisskopf MG (2015) Autism spectrum disorder and particulate matter air pollution before, during, and after pregnancy: a nested case-control analysis within the Nurses’ Health Study II Cohort. Environ Health Perspect 123(3):264–270 25522338 10.1289/ehp.1408133 PMC4348742

[CR43] Ritz B, Liew Z, Yan Q, Cuia X, Virk J, Ketzel M, Raaschou-Nielsen O (2018) Air pollution and autism in Denmark. Environ Epidemiol 2(4):e028 31008439 10.1097/EE9.0000000000000028 PMC6474375

[CR44] Robinson EB, St Pourcain B, Anttila V, Kosmicki JA, Bulik-Sullivan B, Grove J, Maller J, Samocha KE, Sanders SJ, Ripke S (2016) Genetic risk for autism spectrum disorders and neuropsychiatric variation in the general population. Nat Genet 48(5):552–555 26998691 10.1038/ng.3529 PMC4986048

[CR45] Roqué PJ, Dao K, Costa LG (2016) Microglia mediate diesel exhaust particle-induced cerebellar neuronal toxicity through neuroinflammatory mechanisms. Neurotoxicology 56:204–214 27543421 10.1016/j.neuro.2016.08.006 PMC5048600

[CR46] Schafer DP, Stevens B (2015) Brains, blood, and guts: MeCP2 regulates microglia, monocytes, and peripheral macrophages. Immunity 42(4):600–602 25902477 10.1016/j.immuni.2015.04.002

[CR47] Schisterman EF, Perkins NJ, Mumford SL, Ahrens KA, Mitchell EM (2017) Collinearity and causal diagrams: a lesson on the importance of model specification. Epidemiology 28(1):47–53 27676260 10.1097/EDE.0000000000000554 PMC5131787

[CR48] Solomon CG, Willett WC, Carey VJ, Rich-Edwards J, Hunter DJ, Colditz GA, Stampfer MJ, Speizer FE, Spiegelman D, Manson JE (1997) A prospective study of pregravid determinants of gestational diabetes mellitus. JAMA 278(13):1078–1083 9315766

[CR49] Song AY, Feinberg JI, Bakulski KM, Croen LA, Fallin MD, Newschaffer CJ, Hertz-Picciotto I, Schmidt RJ, Ladd-Acosta C, Volk HE (2022) Prenatal exposure to ambient air pollution and epigenetic aging at birth in newborns. Front Genet 13:929416 35836579 10.3389/fgene.2022.929416 PMC9274082

[CR50] Vadillo-Ortega F, Osornio-Vargas A, Buxton MA, Sánchez BN, Rojas-Bracho L, Viveros-Alcaráz M, Castillo-Castrejón M, Beltrán-Montoya J, Brown DG, O’Neill MS (2014) Air pollution, inflammation and preterm birth: a potential mechanistic link. Med Hypotheses 82(2):219–224 24382337 10.1016/j.mehy.2013.11.042 PMC3928635

[CR51] Volk HE, Lurmann F, Penfold B, Hertz-Picciotto I, McConnell R (2013) Traffic-related air pollution, particulate matter, and autism. JAMA Psychiatr 70(1):71–77 10.1001/jamapsychiatry.2013.266 PMC4019010 23404082

[CR52] Wang SY, Cheng YY, Guo HR, Tseng YC (2021) Air pollution during pregnancy and childhood autism spectrum disorder in Taiwan. Int J Environ Res Public Health. 10.3390/ijerph18189784 10.3390/ijerph18189784 PMC8467611 34574710

[CR53] Weisskopf MG, Kioumourtzoglou MA, Roberts AL (2015) Air pollution and autism spectrum disorders: causal or confounded? Curr Environ Health Rep 2(4):430–439 26399256 10.1007/s40572-015-0073-9 PMC4737505

[CR54] Weisskopf MG, Tchetgen Tchetgen EJ, Raz R (2016) Commentary: on the use of imperfect negative control exposures in epidemiologic studies. Epidemiology 27(3):365–367 27035687 10.1097/EDE.0000000000000454 PMC12186581

[CR55] Weisskopf MG, Webster TF (2017) Trade-offs of personal versus more proxy exposure measures in environmental epidemiology. Epidemiology 28(5):635–643 28520644 10.1097/EDE.0000000000000686 PMC5543716

[CR56] Yanosky JD, Paciorek CJ, Schwartz J, Laden F, Puett R, Suh HH (2008) Spatio-temporal modeling of chronic PM10 exposure for the Nurses’ Health Study. Atmos Environ 42(18):4047–4062 10.1016/j.atmosenv.2008.01.044 PMC2705904 19584946

[CR57] Yanosky JD, Paciorek CJ, Suh HH (2009a) Predicting chronic fine and coarse particulate exposures using spatiotemporal models for the Northeastern and Midwestern United States. Environ Health Perspect 117(4):522–529 19440489 10.1289/ehp.11692 PMC2679594

[CR58] Yanosky JD, Paciorek CJ, Suh HH (2008) Predicting chronic fine and coarse particulate exposures using spatiotemporal models for the Northeastern and Midwestern United States. Environ Health Perspect 117(4):522 19440489 10.1289/ehp.11692 PMC2679594

[CR59] Yousefian F, Mahvi AH, Yunesian M, Hassanvand MS, Kashani H, Amini H (2018) Long-term exposure to ambient air pollution and autism spectrum disorder in children: a case-control study in Tehran, Iran. Sci Total Environ 643:1216–1222 30189537 10.1016/j.scitotenv.2018.06.259

[CR60] Zarimeidani F, Rahmati R, Mostafavi M, Darvishi M, Khodadadi S, Mohammadi M, Shamlou F, Bakhtiyari S, Alipourfard I (2025) Gut microbiota and autism spectrum disorder: a neuroinflammatory mediated mechanism of pathogenesis? Inflammation 48(2):501–519 39093342 10.1007/s10753-024-02061-y PMC12053372

[CR61] Zhang Z, Kang W, Mi Y, Zhong X, He Y (2025) The microbiota–gut–brain axis in autism: associations, causal inference, and interventions—a narrative review. Pathogens 14(11):1145 41305382 10.3390/pathogens14111145 PMC12655048

