# Supplementary material for: Perinatal exposure to gaseous pollutants and autism spectrum disorder in children: A nested case–control study in the nurses’ health study ii cohort
Source: Environ Sci Pollut Res Int. 2026 Mar 10;33(11):4753–63. doi: 10.1007/s11356-026-37583-5 (PMC13056788; doi:10.1007/s11356-026-37583-5)
Supplement: Supplementary file 1 — Supplementary file1 (DOCX 411 kb) [file 11356_2026_37583_MOESM1_ESM.docx]

Figure S1. Correlation matrix showing the Pearson correlation coefficients of the air pollutant values from the different 9-month exposure periods: 9 months preconception; 9 months during pregnancy; and first 9 months after birth. Pearson correlation coefficients range from -1 to 1, with values closer to -1 indicating higher negative correlation, values closer to 0 indicating no correlation, and values closer to 1 indicating higher positive correlation.


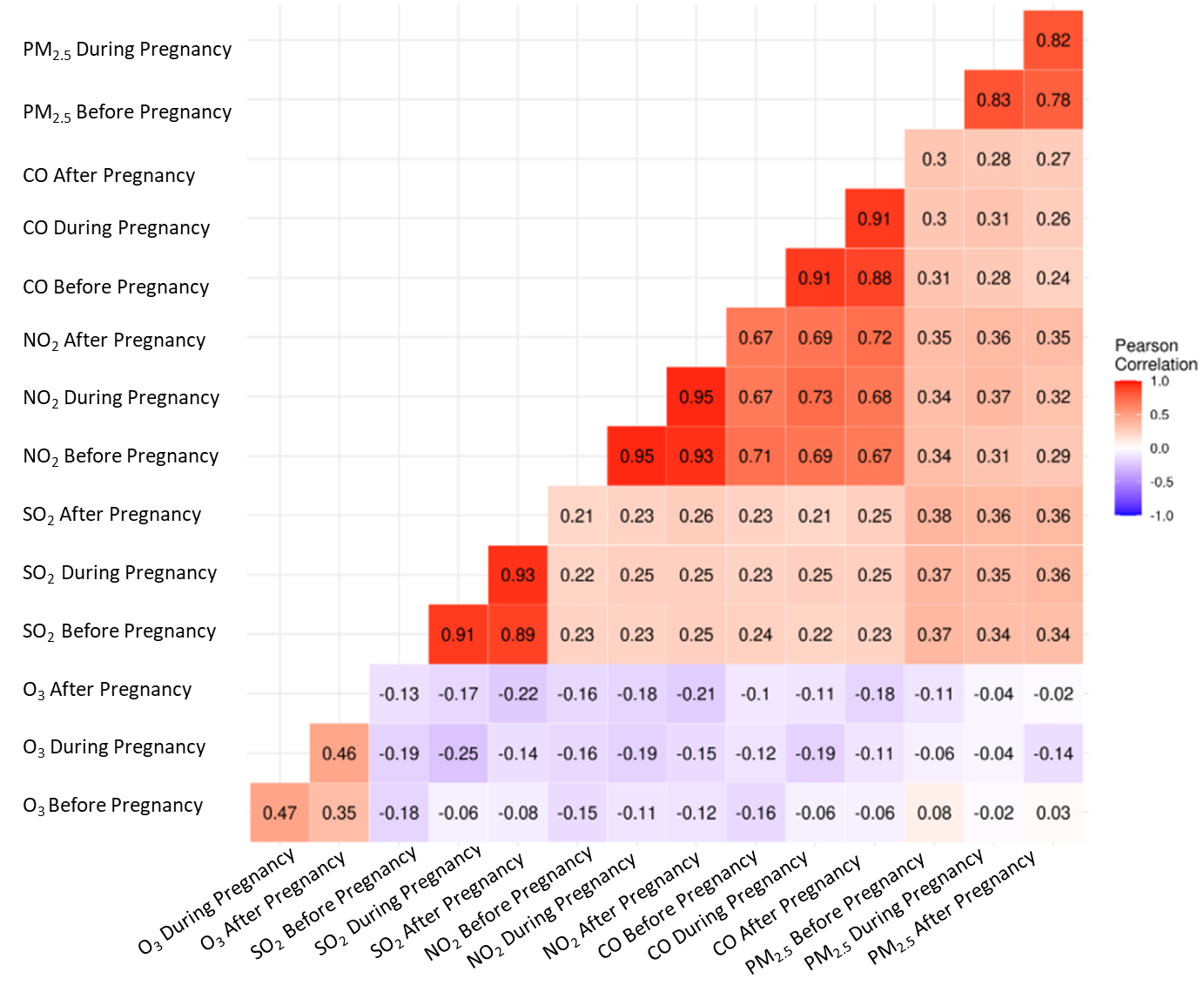


Table S1. Median and interquartile range (IQR) for each air pollutant before, during, and after pregnancy.

|  | Before pregnancy | During pregnancy | After pregnancy |
| --- | --- | --- | --- |
| O_3_ (ppm) | 0.02 (0.020, 0.027) | 0.02 (0.020, 0.027) | 0.02 (0.020, 0.027) |
| SO_2_ (ppb) | 6.26 (3.544, 9.047) | 6.11 (3.425, 8.963) | 5.82 (3.298, 8.352) |
| NO_2_ (ppb) | 18.97 (13.738, 24.271) | 19.06 (13.922, 23.784) | 18.99 (13.982, 23.312) |
| CO (ppm) | 0.91 (0.696, 1.222) | 0.88 (0.664, 1.181) | 0.84 (0.653, 1.135) |

*Note: Values are indicated as median value (IQR).

Table S2. Odds ratios (OR) and 95% confidence intervals (CI) for the association between autism spectrum disorder (ASD) and an interquartile range (IQR) increase in the residentially-linked air pollutants. Effect estimates are shown for the full pregnancy and at potential periods of susceptibility, including 9 months preconception, 1^st^ trimester, 2^nd^ trimester, 3^rd^ trimester, and 9 months post-birth. The associations for the periods of susceptibility were analyzed in separate exposure models and mutually adjusted exposure models.

| Model | Mutually adjusted^*^ |  |
| --- | --- | --- |
| O_3_ (N=1782; cases=250; controls=1532) | | |
| 9 months preconception | 0.80 (0.59, 1.10) | |
| Trimester 1 | 1.01 (0.79, 1.30) | |
| Trimester 2 | 1.12 (0.95, 1.32) | |
| Trimester 3 | 0.95 (0.76, 1.20) | |
| 9 months after birth | 1.08 (0.75, 1.57) | |
| SO_2_ (N=1770; cases=250; controls=1520) | | |
| 9 months preconception | 2.92 (1.60, 5.33) | |
| Trimester 1 | 0.84 (0.52, 1.35) | |
| Trimester 2 | 0.79 (0.53, 1.18) | |
| Trimester 3 | 0.64 (0.38, 1.05) | |
| 9 months after birth | 0.86 (0.40, 1.83) | |
| NO_2_ (N=1731; cases=244; controls=1487) | | |
| 9 months preconception | 0.84 (0.48, 1.47) | |
| Trimester 1 | 0.99 (0.58, 1.71) | |
| Trimester 2 | 1.06 (0.67, 1.68) | |
| Trimester 3 | 1.97 (1.11, 3.50) | |
| 9 months after birth | 0.75 (0.39, 1.45) | |
| CO (N=1765; cases=249; controls=1516) | | |
| 9 months preconception | 0.97 (0.59, 1.60) | |
| Trimester 1 | 0.71 (0.45, 1.11) | |
| Trimester 2 | 1.20 (0.86, 1.67) | |
| Trimester 3 | 1.11 (0.69, 1.78) | |
| 9 months after birth | 1.49 (0.81, 2.75) | |

**Mutually adjusted for the other two exposure periods for the same air pollutants, as well as all other covariates (child sex, year of birth, month of birth, maternal age at birth, paternal age at birth, and census income.)*

*IQR: O_3_ (0.007 ppm); SO_2_ (5.54 ppb); NO_2_ (9.86 ppb); CO (0.52 ppm)*

Table S3. Odds ratios (OR) and 95% confidence intervals (CI) for the association between autism spectrum disorder (ASD) and an interquartile range (IQR) increase in the residentially-linked air pollutants. Effect estimates were shown for the full pregnancy and at potential periods of susceptibility, including 9 months and 3 months preconception, 9 months during pregnancy, 1^st^ trimester, 2^nd^ trimester, 3^rd^ trimester, and 9 months and 3 months post-birth. The associations for the periods of susceptibility were analyzed in separate exposure models and mutually adjusted exposure models. The population was restricted to those with exposure datasets for the specific pollutant and PM_2.5_.

|  |  | O_3_ (N=1762; cases=247; controls=1515) | | SO_2_ (N=1750; cases=247; controls=1503) | | NO_2_ (N=1712; cases=241; controls=1471) | | CO (N=1746; cases=246; controls=1500) | |
| --- | --- | --- | --- | --- | --- | --- | --- | --- | --- |
| Model | | Adjusted^*^ | Mutually adjusted^**^ | Adjusted^*^ | Mutually adjusted^**^ | Adjusted^*^ | Mutually adjusted^**^ | Adjusted^*^ | Mutually adjusted^**^ |
| Before conception | 9 months | 0.73 (0.59, 0.91) | 0.76 (0.60, 0.95) | 1.13 (0.87, 1.47) | 1.12 (0.86, 1.46) | 0.99 (0.60, 1.64) | 1.25 (0.73, 2.14) | 0.99 (0.64, 1.54) | 1.05 (0.67, 1.64) |
|  | 3 months | 0.97 (0.73, 1.29) | 0.98 (0.74, 1.31) | 1.53 (0.91, 2.60) | 1.44 (0.83, 2.52) | 0.89 (0.52, 1.52) | 0.92 (0.53, 1.59) | 0.90 (0.57, 1.43) | 0.88 (0.55, 1.40) |
| During pregnancy | whole pregnancy | 1.11 (0.83, 1.47) | 1.06 (0.79, 1.44) | 0.53 (0.26, 1.10) | 0.58 (0.28, 1.20) | 1.63 (0.86, 3.09) | 1.19 (0.61, 2.34) | 1.20 (0.70, 2.05) | 0.93 (0.53, 1.62) |
|  | Trimester 1 | 0.98 (0.79, 1.22) | 0.98 (0.79, 1.23) | 0.71 (0.47, 1.06) | 0.63 (0.40, 1.01) | 0.93 (0.54, 1.61) | 0.90 (0.50, 1.60) | 0.79 (0.52, 1.19) | 0.82 (0.54, 1.26) |
|  | Trimester 2 | 1.18 (0.94, 1.48) | 1.18 (0.94, 1.49) | 1.21 (0.80, 1.85) | 1.48 (0.90, 2.45) | 0.95 (0.54, 1.66) | 0.91 (0.50, 1.65) | 1.35 (0.89, 2.04) | 1.26 (0.82, 1.93) |
|  | Trimester 3 | 0.75 (0.55, 1.02) | 0.75 (0.55, 1.03) | 0.74 (0.40, 1.38) | 0.58 (0.29, 1.17) | 2.01 (0.99, 4.11) | 1.83 (0.87, 3.82) | 0.92 (0.50, 1.71) | 0.81 (0.43, 1.54) |
| After birth | 9 months | 1.13 (0.87, 1.47) | 1.12 (0.86, 1.46) | 0.89 (0.45, 1.76) | 0.78 (0.39, 1.57) | 0.82 (0.45, 1.48) | 0.86 (0.45, 1.61) | 1.14 (0.67, 1.92) | 1.34 (0.77, 2.31) |
|  | 3 months | 1.18 (0.83, 1.68) | 1.20 (0.84, 1.71) | 1.10 (0.53, 2.31) | 1.30 (0.59, 2.84) | 0.83 (0.42, 1.64) | 0.94 (0.46, 1.90) | 1.50 (0.77, 2.93) | 1.73 (0.87, 3.47) |

**ORs are adjusted for child sex, year of birth, month of birth, maternal age at birth, paternal age at birth, census income, and mutually adjusted for the other two exposure periods for the same air pollutants.*

***Mutually adjusted for the other two exposure periods for the same air pollutants and PM_2.5_, as well as all other covariates listed above.*

*IQR: O_3_ (0.007 ppm); SO_2_ (5.54 ppb); NO_2_ (9.86 ppb); CO (0.52 ppm)*

Table S4. Odds ratios (OR) and 95% confidence intervals (CI) for the association between autism spectrum disorder (ASD) and an interquartile range (IQR) increase in the residentially-linked air pollutants by different sex. Effect estimates are shown for the full pregnancy and at potential periods of susceptibility, including 9 months preconception, 9 months during pregnancy, and 9 months post-birth. The associations for the periods of susceptibility were analyzed in separate exposure models and mutually adjusted exposure models.

|  | Male population | | | Female population | | |
| --- | --- | --- | --- | --- | --- | --- |
| Model | Unadjusted | Adjusted^*^ | Mutually adjusted^**^ | Unadjusted | Adjusted^*^ | Mutually adjusted^**^ |
|  | O_3_ (N=1005; cases=211; controls=794) | | | O_3_ (N=777; cases=39; controls=738) | | |
| 9 months before conception | 0.74 (0.60, 0.90) | 0.74 (0.61, 0.91) | 0.67 (0.53, 0.86) | 1.09 (0.71, 1.68) | 1.06 (0.69, 1.63) | 1.04 (0.64, 1.71) |
| Whole pregnancy | 0.95 (0.77, 1.16) | 0.98 (0.78, 1.24) | 1.11 (0.80, 1.52) | 1.00 (0.64, 1.56) | 1.08 (0.66, 1.76) | 1.10 (0.57, 2.14) |
| 9 months after birth | 1.04 (0.84, 1.28) | 1.06 (0.84, 1.32) | 1.17 (0.88, 1.57) | 1.00 (0.61, 1.61) | 1.00 (0.61, 1.61) | 0.93 (0.51, 1.69) |
|  | SO_2_ (N=997; cases=211; controls=786) | | | SO_2_ (N=773; cases=39; controls=734) | | |
| 9 months before conception | 1.24 (1.00, 1.54) | 1.25 (1.00, 1.57) | 2.47 (1.36, 4.47) | 0.97 (0.62, 1.52) | 0.98 (0.61, 1.58) | 0.79 (0.17, 3.72) |
| Whole pregnancy | 1.09 (0.88, 1.36) | 1.09 (0.87, 1.38) | 0.46 (0.20, 1.04) | 0.91 (0.58, 1.44) | 0.90 (0.55, 1.48) | 0.68 (0.16, 2.84) |
| 9 months after birth | 1.12 (0.88, 1.42) | 1.13 (0.88, 1.45) | 1.01 (0.46, 2.21) | 0.82 (0.49, 1.36) | 0.86 (0.50, 1.49) | 1.61 (0.51, 5.10) |
|  | NO_2_ (N=977; cases=206; controls=771) | | | NO_2_ (N=754; cases=38; controls=716) | | |
| 9 months before conception | 1.32 (1.12, 1.55) | 1.35 (1.13, 1.60) | 0.99 (0.57, 1.73) | 1.15 (0.80, 1.65) | 1.26 (0.86, 1.85) | 0.98 (0.28, 3.44) |
| Whole pregnancy | 1.34 (1.14, 1.58) | 1.37 (1.15, 1.63) | 1.63 (0.80, 3.31) | 1.19 (0.82, 1.75) | 1.32 (0.89, 1.96) | 2.00 (0.46, 8.71) |
| 9 months after birth | 1.33 (1.12, 1.58) | 1.35 (1.12, 1.62) | 0.84 (0.44, 1.62) | 1.25 (0.83, 1.89) | 1.25 (0.83, 1.89) | 0.64 (0.16, 2.61) |
|  | CO (N=997; cases=210; controls=787) | | | CO (N=768; cases=39; controls=729) | | |
| 9 months before conception | 1.23 (1.02, 1.49) | 1.30 (1.05, 1.62) | 1.20 (0.74, 1.95) | 1.17 (0.80, 1.73) | 1.41 (0.91, 2.18) | 0.68 (0.25, 1.81) |
| Whole pregnancy | 1.23 (1.02, 1.48) | 1.28 (1.03, 1.59) | 0.99 (0.54, 1.80) | 1.34 (0.91, 1.99) | 1.63 (1.04, 2.57) | 1.96 (0.63, 6.13) |
| 9 months after birth | 1.24 (1.01, 1.51) | 1.31 (1.04, 1.65) | 1.12 (0.63, 2.01) | 1.26 (0.83, 1.92) | 1.61 (0.99, 2.61) | 1.23 (0.37, 4.13) |

**ORs are adjusted for child sex, year of birth, month of birth, maternal age at birth, paternal age at birth, and census income.*

***Mutually adjusted for the other two exposure periods for the same air pollutants, as well as all other covariates listed above.*

*IQR: O_3_ (0.007 ppm); SO_2_ (5.54 ppb); NO_2_ (9.86 ppb); CO (0.52 ppm)*

Table S5. Odds ratios (OR) and 95% confidence intervals (CI) for the association between autism spectrum disorder (ASD) and an interquartile range (IQR) increase in the residentially-linked air pollutants by different sex. Effect estimates are shown for the full pregnancy and at potential periods of susceptibility, including 3 months preconception, 1^st^ trimester, 2^nd^ trimester, 3^rd^ trimester, and 3 months post-birth. The associations for the periods of susceptibility were analyzed in separate exposure models and mutually adjusted exposure models.

|  | Male population | | | Female population | | |
| --- | --- | --- | --- | --- | --- | --- |
|  | O_3_ (N=1005; cases=211; controls=794) | | | O_3_ (N=777; cases=39; controls=738) | | |
| 3 months preconception | 0.95 (0.85, 1.07) | 0.89 (0.77, 1.04) | 0.95 (0.69, 1.31) | 1.21 (0.93, 1.57) | 1.08 (0.77, 1.51) | 1.04 (0.57, 1.90) |
| Trimester 1 | 1.13 (1.01, 1.28) | 1.15 (1.02, 1.31) | 0.95 (0.75, 1.21) | 1.08 (0.84, 1.39) | 0.96 (0.73, 1.27) | 1.03 (0.64, 1.68) |
| Trimester 2 | 1.01 (0.90, 1.13) | 1.07 (0.92, 1.24) | 1.23 (0.95, 1.58) | 0.93 (0.72, 1.21) | 1.05 (0.76, 1.46) | 1.07 (0.63, 1.83) |
| Trimester 3 | 0.81 (0.72, 0.92) | 0.81 (0.71, 0.92) | 0.68 (0.48, 0.96) | 1.02 (0.79, 1.32) | 1.10 (0.84, 1.45) | 1.12 (0.57, 2.21) |
| 3 months after birth | 0.89 (0.79, 1.00) | 0.84 (0.73, 0.96) | 1.23 (0.82, 1.84) | 1.10 (0.85, 1.43) | 1.06 (0.80, 1.40) | 1.02 (0.47, 2.18) |
|  | SO_2_ (N=997; cases=211; controls=786) | | | SO_2_ (N=773; cases=39; controls=734) | | |
| 3 months preconception | 1.18 (0.97, 1.43) | 1.23 (1.00, 1.52) | 1.60 (0.89, 2.87) | 0.95 (0.63, 1.44) | 1.03 (0.65, 1.61) | 1.90 (0.59, 6.14) |
| Trimester 1 | 1.05 (0.87, 1.27) | 1.06 (0.86, 1.29) | 0.75 (0.48, 1.15) | 0.83 (0.54, 1.26) | 0.88 (0.56, 1.39) | 0.70 (0.25, 1.99) |
| Trimester 2 | 1.08 (0.89, 1.31) | 1.07 (0.87, 1.32) | 1.21 (0.76, 1.91) | 0.96 (0.64, 1.42) | 0.92 (0.60, 1.42) | 0.98 (0.32, 3.00) |
| Trimester 3 | 1.12 (0.91, 1.37) | 1.10 (0.88, 1.36) | 0.72 (0.37, 1.41) | 0.96 (0.62, 1.51) | 0.95 (0.59, 1.53) | 0.96 (0.20, 4.67) |
| 3 months after birth | 1.15 (0.93, 1.41) | 1.17 (0.94, 1.45) | 1.09 (0.49, 2.46) | 1.01 (0.65, 1.57) | 1.01 (0.63, 1.61) | 0.72 (0.13, 4.10) |
|  | NO_2_ (N=977; cases=206; controls=771) | | | NO_2_ (N=754; cases=38; controls=716) | | |
| 3 months preconception | 1.27 (1.09, 1.49) | 1.30 (1.10, 1.53) | 0.91 (0.51, 1.65) | 1.03 (0.72, 1.47) | 1.17 (0.81, 1.70) | 0.74 (0.18, 2.96) |
| Trimester 1 | 1.25 (1.07, 1.46) | 1.26 (1.07, 1.48) | 0.97 (0.54, 1.75) | 1.12 (0.77, 1.62) | 1.25 (0.86, 1.84) | 0.85 (0.20, 3.68) |
| Trimester 2 | 1.30 (1.12, 1.51) | 1.32 (1.12, 1.56) | 0.87 (0.47, 1.61) | 1.22 (0.85, 1.74) | 1.31 (0.90, 1.91) | 1.58 (0.38, 6.54) |
| Trimester 3 | 1.39 (1.18, 1.64) | 1.43 (1.20, 1.70) | 2.51 (1.15, 5.49) | 1.08 (0.74, 1.58) | 1.21 (0.81, 1.81) | 0.62 (0.10, 3.86) |
| 3 months after birth | 1.33 (1.12, 1.56) | 1.36 (1.14, 1.62) | 0.71 (0.34, 1.49) | 1.13 (0.78, 1.65) | 1.28 (0.87, 1.90) | 2.01 (0.39, 10.41) |
|  | CO (N=997; cases=210; controls=787) | | | CO (N=768; cases=39; controls=729) | | |
| 3 months preconception | 1.15 (0.97, 1.36) | 1.22 (1.00, 1.48) | 1.15 (0.69, 1.89) | 0.99 (0.69, 1.43) | 1.24 (0.82, 1.88) | 0.47 (0.14, 1.55) |
| Trimester 1 | 1.11 (0.94, 1.31) | 1.11 (0.92, 1.34) | 0.72 (0.47, 1.12) | 1.14 (0.78, 1.65) | 1.40 (0.92, 2.13) | 1.45 (0.46, 4.55) |
| Trimester 2 | 1.22 (1.03, 1.43) | 1.25 (1.04, 1.50) | 1.42 (0.91, 2.21) | 1.33 (0.94, 1.87) | 1.52 (1.02, 2.27) | 0.92 (0.29, 2.95) |
| Trimester 3 | 1.25 (1.04, 1.49) | 1.30 (1.06, 1.59) | 0.89 (0.46, 1.72) | 1.29 (0.88, 1.90) | 1.55 (1.00, 2.40) | 1.25 (0.25, 6.22) |
| 3 months after birth | 1.20 (1.00, 1.44) | 1.30 (1.05, 1.60) | 1.25 (0.60, 2.58) | 1.29 (0.88, 1.87) | 1.60 (1.04, 2.48) | 2.06 (0.41, 10.32) |

**ORs are adjusted for child sex, year of birth, month of birth, maternal age at birth, paternal age at birth, and census income.*

***Mutually adjusted for the other two exposure periods for the same air pollutants, as well as all other covariates listed above.*

*IQR: O_3_ (0.007 ppm); SO_2_ (5.54 ppb); NO_2_ (9.86 ppb); CO (0.52 ppm)*

Table S6. Odds ratios (OR) and 95% confidence intervals (CI) for the association between autism spectrum disorder (ASD) and an interquartile range (IQR) increase in the residentially-linked air pollutants. Effect estimates are shown for the full pregnancy and at potential periods of susceptibility, including 3 months preconception, 1^st^ trimester, 2^nd^ trimester, 3^rd^ trimester, and 3 months post-birth. The associations for the periods of susceptibility were analyzed in separate exposure models and mutually adjusted exposure models. The population was restricted to the non-mover population only.

| Model | Mutually adjusted^*^ | Mutually adjusted for PM_2.5_^**^ |
| --- | --- | --- |
| O_3_ (N=1160; cases=165; controls=995) | |  |
| 3 months preconception | 0.96 (0.67, 1.38) | 0.97 (0.67, 1.40) |
| Trimester 1 | 1.02 (0.79, 1.33) | 1.06 (0.80, 1.39) |
| Trimester 2 | 1.09 (0.82, 1.44) | 1.04 (0.77, 1.39) |
| Trimester 3 | 0.83 (0.57, 1.21) | 0.83 (0.56, 1.22) |
| 3 months after birth | 1.02 (0.65, 1.62) | 1.07 (0.67, 1.70) |
| SO_2_ (N=1151; cases=165; controls=986) | |  |
| 3 months preconception | 2.00 (1.02, 3.89) | 1.63 (0.79, 3.35) |
| Trimester 1 | 0.83 (0.51, 1.35) | 0.66 (0.36, 1.19) |
| Trimester 2 | 0.87 (0.50, 1.52) | 1.19 (0.61, 2.33) |
| Trimester 3 | 0.82 (0.37, 1.80) | 0.60 (0.24, 1.50) |
| 3 months after birth | 0.84 (0.32, 2.16) | 1.16 (0.42, 3.23) |
| NO_2_ (N=1120; cases=161; controls=959) | |  |
| 3 months preconception | 1.10 (0.54, 2.24) | 1.13 (0.54, 2.35) |
| Trimester 1 | 0.91 (0.47, 1.77) | 0.88 (0.43, 1.81) |
| Trimester 2 | 0.82 (0.41, 1.64) | 0.79 (0.37, 1.67) |
| Trimester 3 | 3.71 (1.47, 9.35) | 3.00 (1.15, 7.83) |
| 3 months after birth | 0.46 (0.19, 1.15) | 0.56 (0.22, 1.45) |
| CO (N=1145; cases=164; controls=981) | |  |
| 3 months preconception | 0.97 (0.54, 1.75) | 0.81 (0.44, 1.50) |
| Trimester 1 | 0.71 (0.43, 1.20) | 0.78 (0.44, 1.36) |
| Trimester 2 | 1.24 (0.75, 2.04) | 1.18 (0.69, 2.01) |
| Trimester 3 | 1.03 (0.47, 2.25) | 0.72 (0.31, 1.69) |
| 3 months after birth | 1.52 (0.64, 3.61) | 2.35 (0.91, 6.05) |

**Mutually adjusted for the other two exposure periods for the same air pollutants, as well as all other covariates (child sex, year of birth, month of birth, maternal age at birth, paternal age at birth, and census income).*

***Mutually adjusted for the other two exposure periods for the same air pollutants and PM_2.5_, as well as all other covariates listed above.*

*IQR: O_3_ (0.007 ppm); SO_2_ (5.54 ppb); NO_2_ (9.86 ppb); CO (0.52 ppm)*
